# Genes involved in *Drosophila *glutamate receptor expression and localization

**DOI:** 10.1186/1471-2202-6-44

**Published:** 2005-06-28

**Authors:** Faith LW Liebl, David E Featherstone

**Affiliations:** 1 Dept. of Biological Sciences, University of Illinois at Chicago, Chicago, USA; 2 Dept. of Cell and Structural Biology, Univ. of Illinois at Urbana-Champaign, Urbana, USA

## Abstract

**Background:**

A clear picture of the mechanisms controlling glutamate receptor expression, localization, and stability remains elusive, possibly due to an incomplete understanding of the proteins involved. We screened transposon mutants generated by the ongoing *Drosophila *Gene Disruption Project in an effort to identify the different types of genes required for glutamate receptor cluster development.

**Results:**

To enrich for non-silent insertions with severe disruptions in glutamate receptor clustering, we identified and focused on homozygous lethal mutants in a collection of 2185 BG and KG transposon mutants generated by the BDGP Gene Disruption Project. 202 lethal mutant lines were individually dissected to expose glutamatergic neuromuscular junctions, stained using antibodies that recognize neuronal membrane and the glutamate receptor subunit GluRIIA, and viewed using laser-scanning confocal microscopy. We identified 57 mutants with qualitative differences in GluRIIA expression and/or localization. 84% of mutants showed loss of receptors and/or clusters; 16% of mutants showed an increase in receptors. Insertion loci encode a variety of protein types, including cytoskeleton proteins and regulators, kinases, phosphatases, ubiquitin ligases, mucins, cell adhesion proteins, transporters, proteins controlling gene expression and protein translation, and proteins of unknown/novel function. Expression pattern analyses and complementation tests, however, suggest that any single mutant – even if a mutant gene is uniquely tagged – must be interpreted with caution until the mutation is validated genetically and phenotypically.

**Conclusion:**

Our study identified 57 transposon mutants with qualitative differences in glutamate receptor expression and localization. Despite transposon tagging of every insertion locus, extensive validation is needed before one can have confidence in the role of any individual gene. Alternatively, one can focus on the *types *of genes identified, rather than the identities of individual genes. This genomic approach, which circumvents many technical caveats in favor of a wider perspective, suggests that glutamate receptor cluster formation involves many cellular processes, including: 1) cell adhesion and signaling, 2) extensive and relatively specific regulation of gene expression and RNA, 3) the actin and microtubule cytoskeletons, and 4) many novel/unexplored processes, such as those involving mucin/polycystin-like proteins and proteins of unknown function.

## Background

The vast majority of fast synaptic transmission in the mammalian central nervous system is glutamatergic. Proper expression, localization, and regulation of glutamate receptors are critical for brain development and plasticity. Not surprisingly, the molecular mechanisms controlling glutamate receptor expression, localization, and stability are of great interest. The most common recent approach to understanding these mechanisms has been biochemical: Proteins are identified based on a biochemical interaction with a glutamate receptor subunit. This approach has identified a number of important candidates, some of which have been subsequently shown to play important roles in glutamate receptor trafficking and stability at the PSD (for review see [1,2]).

An alternative approach is forward genetics: Mutant animals are screened for alterations in glutamate receptor localization. Perhaps the most well-known glutamate receptor trafficking protein identified via genetics is stargazin, which is disrupted in stargazer mutant mice [3]. The identification of stargazin was serendipitous; mammalian genetic screens are unfortunately still relatively time-consuming and expensive (though this may change as RNAi techniques advance). Most forward genetic screens glutamate receptor mutants have used *C. elegans*. Studies using *C. elegans *have highlighted the importance of ubiquitination, CamKII, and PDZ proteins in controlling glutamate receptor number, and identified a novel protein required for nematode glutamate receptor function [4-7]. Unfortunately, glutamatergic synapses in *C. elegans *are accessible for electrophysiology only with great difficulty. The subunit composition of *C. elegans *receptors in vivo has also not yet been determined, which hinders the study of subunit-specific trafficking mechanisms.

*Drosophila *are not only amenable to powerful genetics, but also contain glutamatergic neuromuscular junctions (NMJs) that are individually identifiable and accessible throughout development to high-resolution electrophysiological and microscopic techniques. These techniques have revealed a significant amount of information concerning this popular model synapse, including the exact subunit composition of glutamate receptors in vivo. *Drosophila *NMJs contain two subtypes of postsynaptic glutamate receptor, which are molecularly, pharmacologically, and spatially distinct [8-11]. The 'A' receptor subtype contains the subunit GluRIIA, in combination with subunits GluRIIC, GluRIID, and GluRIIE. The 'B' receptor subtype contains the subunit GluRIIB, in combination with the subunits GluRIIC, GluRIID, and GluRIIE. Both receptor subtypes are most similar in sequence to mammalian kainate receptors. The *Drosophila *genome also encodes receptor subunits with high similarity to mammalian AMPA, delta, and NMDA receptor subunits, but these proteins are not found postsynaptically in the NMJ. Virtually nothing is known about the molecular mechanisms that control *Drosophila *glutamate receptor expression, localization, and stability.

To determine the molecular mechanisms controlling *Drosophila *glutamate receptor expression, localization, and stability, we are screening transposon insertion mutants generated by the Berkeley *Drosophila *Gene Disruption Project [12]. This mutant collection contains insertions in over 40% of the entire genome, and the insertion site for almost every mutant has already been identified by inverse PCR. With the addition of transposon insertions from other collections, it is likely that Drosophilists will have access to insertion mutants of almost every gene in the fly genome within a few years. Theoretically, one could quickly and efficiently identify the complete list of genes required for any particular process simply by examining a non-redundant set of mutants for a phenotype of interest. We are testing this idea and using this approach to define the broad categories of genes required for glutamate receptor cluster formation. We identified 57 lethal mutants with qualitatively abnormal glutamate receptor clusters. Here, we present this list and discuss the types of genes that are, and are not, represented. Because of the high prevalence of background mutations even in transposon mutant collections, the role of individual genes must be extensively confirmed before a role for any particular protein is assumed. However, this problem may be circumvented, and perhaps more insight gained, by focusing on the types of genes identified rather than the identity of individual genes. We do so here.

## Results

We sought to identify *Drosophila *mutants with abnormal glutamate receptor cluster development. Elimination of *Drosophila *NMJ glutamate receptors results in paralysis and embryonic lethality [9]. Mutations that reduce (but do not eliminate) NMJ glutamate receptors allow hatching, but typically cause larval or pupal lethality [8-10]. Any effective screen for glutamate receptor cluster formation mutants must therefore include examination of homozygous lethal mutants. However, proper examination of glutamate receptor clusters in embryos and larvae requires technically challenging dissection techniques and time-consuming confocal microscopy. Therefore, we sought to minimize examination of mutants that do not have glutamate receptor cluster defects. To do this, we made the assumption, based on the studies cited above, that mutants with severe defects in glutamate receptor cluster formation are more likely to be homozygous lethal. Thus, the first step in our screen for mutants with reduced or eliminated glutamate receptors was identification of recessive lethal transposon mutants (e.g. insertions lines with no homozygous viable adults). We concentrated on the collection of *GT1 *and *SuPor-P *insertion mutants, since these transposons were engineered for maximal gene disruption [13,14]. Of 2185 mutants (representing insertions in approximately 16% of the entire genome), 220 insertion lines contained lethal mutations. Because a prerequisite for NMJ glutamate cluster formation is development of neuromusculature, we examined dechorionated embryos from each of the 220 stocks to ensure that homozygous mutants developed into 'morphologically mature' late stage embryos typical of wildtype animals 16–17 hr after egg laying, AEL, which is when NMJs begin to form [15-18]. Morphological maturity was based on the presence of characteristics typical of late stage 17: clear segmentation, mouthhooks, condensed CNS, malphigian tubules, and visible trachea. 205 of 220 (93%) of the homozygous lethal mutants formed morphologically mature embryos. 202 of these mutants were rebalanced using a GFP-tagged balancer chromosome for unambiguous identification of homozygous mutant animals. We were unable to rebalance 3 mutant lines using chromosome-appropriate GFP-tagged balancers, possibly because the insertion mutant chromosome and balancer chromosome both contained lethal mutations that fail to complement. We then verified that all 202 GFP-balanced stocks did indeed carry recessive lethal mutations and determined the latest stage to which homozygous (non GFP) embryos and larvae lived.

**Figure 1:**
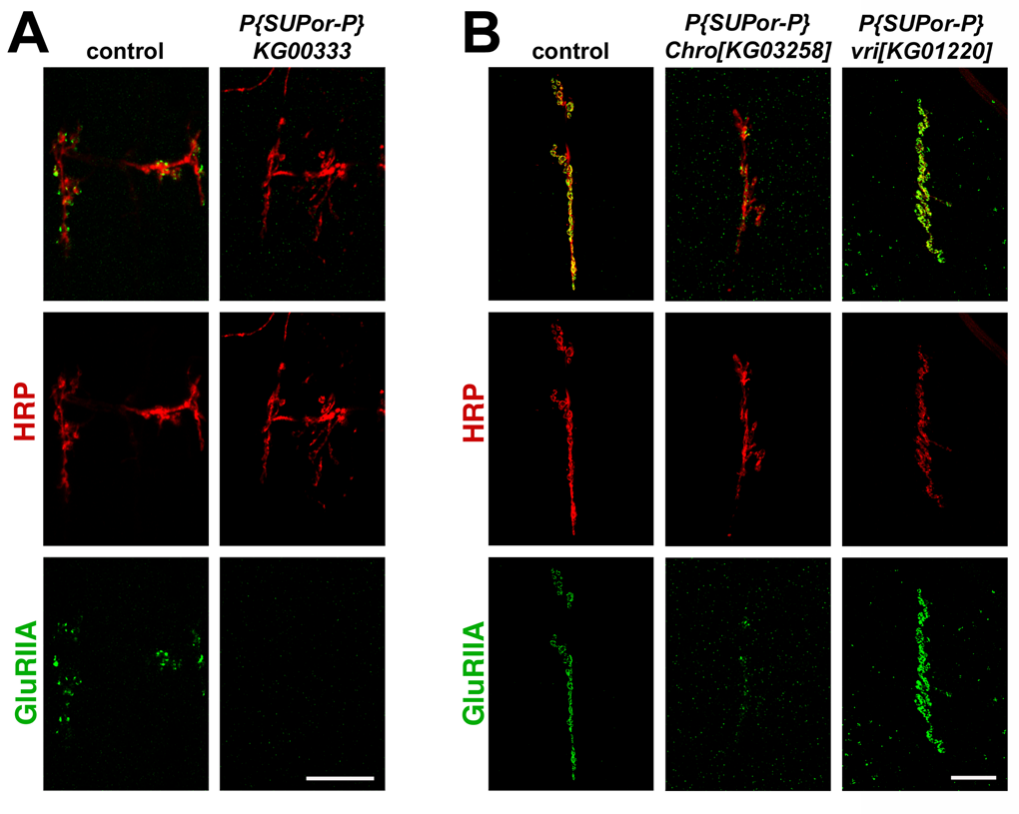
**Example phenotypes identified in the screen. **A: NMJs on embryonic ventral longitudinal muscles 7, 6, and 13, visualized using anti-HRP antibodies (which stain all neuronal membrane) and anti-GluRIIA antibodies (which stain postsynaptic glutamate receptors). HRP immunoreactivity is red; GluRIIA immunoreactivity is green. In wildtype NMJs (A, left column), GluRIIA immunoreactivity appears as puncta, representing clusters of postsynaptic glutamate receptors. In homozygous *P{SUPor-P}KG00333 *mutants (A, right column), GluRIIA immunoreactivity is eliminated. B: NMJs on third instar larval longitudinal muscles 7 and 6, visualized using anti-HRP and anti-GluRIIA antibodies. In wildtype NMJs (B. left column), GluRIIA immunoreactivity appears as large blobs, representing developmentally merged puncta, at sites of innervation. In homozygous *P{SUPor-P}Chro [KG03258] *mutants (B, middle column), synaptic GluRIIA immunoreactivity is dramatically decreased, although extrasynaptic receptors remain prominent. In contrast, synaptic GluRIIA immunoreactivity is dramatically increased in homozygous *P{SUPor-P}vri [KG01220] *mutants (B, right column). Scale bars: 10 micrometers.

For each of the 202 balanced lines, several (typically 5–10) homozygous mutant animals, along with control heterozygous siblings, were manually dissected at the latest viable stage to expose NMJs on ventral longitudinal muscles, then fixed and stained using anti-HRP antibodies that recognize all neuronal membrane (including peripheral axons and NMJs) and anti-GluRIIA antibodies. After dissection, fixation, and staining, each of the 202 rebalanced mutant lines was examined for changes in NMJ morphology and glutamate receptor expression using laser-scanning confocal fluorescent microscopy. 57 of the 202 rebalanced lethal mutants (28%) displayed consistent (most NMJs in several animals) defects in glutamate receptor expression without severe presynaptic morphological abnormalities (e.g. 57 mutants were identified in which NMJs formed, but glutamate receptor clusters were altered). Examples of some of these phenotypes, from both embryonic and larval NMJs, are shown in Fig. 1.

All phenotypes could be classified into one of two broad categories: 1) loss of glutamate receptors (fewer glutamate receptor clusters or smaller individual clusters), or 2) gain of glutamate receptors (more clusters or larger clusters). We relied on cluster size and number because cluster size and number measurements (compared to fluorescence intensity measurements) avoid a requirement for fluorescence intensity calibration or problems associated with potential differences in background immunofluorescence between genotypes. Assuming constant receptor density, cluster size should be directly proportional to the number of clustered receptors. In support of this assumption, immunoreactive cluster sizes correlate well with high-resolution patch-clamp electrophysiological measurements between genotypes [11,19,20] and throughout embryonic/larval NMJ development (Featherstone, unpublished observations).

Most (84%) of the mutants with disruptions in glutamate receptor clusters showed a qualitative loss in receptors. Severe loss of GluRIIA was always associated with embryonic/L1 lethality. One example is *P{SUPor-P}KG00333*, which shows a complete loss of A-type receptors despite the presence of morphologically normal presynaptic terminals (Fig. 1A, left). Some mutants with loss of receptors survived until pupation. An example of a third instar viable mutant, *P{SUPor-P} Chro*^*KG03258*^, with reduced GluRIIA is shown in Fig. 1B (middle column).

A minority (16%) of third instar viable mutants showed a qualitative increase in GluRIIA immunoreactivity. An example of one of these mutants, *P{SUPor-P} vri*^*KG01220*^, is shown in Fig. 1B (right column). Except for *P{SUPor-P}KG00212*, all of the mutants that displayed an increase in GluRIIA immunoreactivity were viable as third instar larvae and also displayed an increase in the number of presynaptic boutons. This presynaptic phenotype is consistent with previous *Drosophila *studies showing that overexpression of postsynaptic GluRIIA causes presynaptic overgrowth [21]. BG and KG transposons are designed to generate loss of function mutations. Therefore, the isolation of mutants with increased receptor cluster size suggests that receptor insertion and clustering per se are not rate-limiting. There are molecular mechanisms, revealed by our screen, that actively restrain the number of synaptic glutamate receptors.

**Figure 2:**
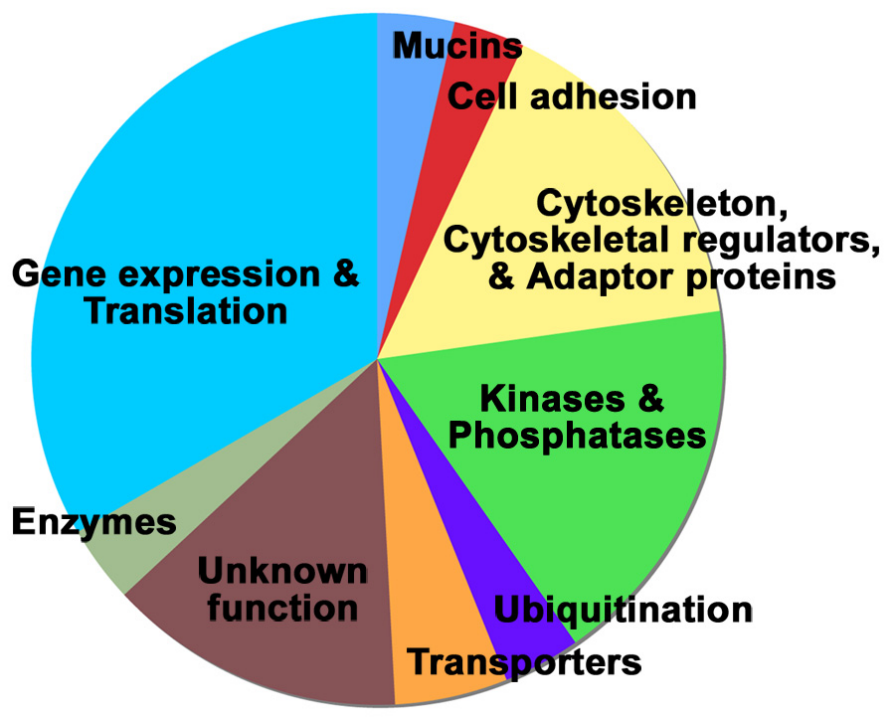
**Pie chart showing the different types of proteins encoded by the insertion loci identified in the screen. **The size of the pie wedge represents the proportion of genes of each type identified. Functional categories were based on previously published studies of the *Drosophila *gene and/or sequence similarity to functionally annotated mouse genes. Table 1 contains a complete list of the genes represented in this chart.

For all of the mutants identified in our screen, the Gene Disruption Project has precisely determined the genomic insertion site of the transposon using inverse PCR. In the vast majority of cases, inverse PCR results were consistent with a unique insertion, and in most cases the flanking genomic sequence revealed that the insertion was in an annotated gene. For each of the mutants identified in our screen, we used the BDGDP *P*-screen database  and/or BLAST searches with flanking genomic sequence from iPCR results to identify which gene was mutated by the inserted *P*-element. Putative functions were assigned to each of these genes based on previous publications, FlyBase annotations, and/or Genbank BLAST searches. We binned each of the insertion site genes into one of the following functional categories: 1) extracellular matrix proteins (mucins), 2) cell adhesion proteins 3) cytoskeleton proteins, cytoskeletal regulators, and adaptor proteins, 4) kinases and phosphatases, 5) ubiqitination proteins (ubiquitin ligases), 6) transporters/pumps, 7) proteins involved in gene expression and protein translation, 8) enzymes, and 9) proteins of unknown/novel function. These categories, and the relative number of proteins in each category, are illustrated by Figure 2. All categories shown in Figure 2 were represented by at least two mutant genes.

Table 1 contains the complete list of all mutants identified in our screen, listed by functional category. For each mutant, Table 1 indicates whether the phenotype was loss (down arrow) or increase (up arrow) in postsynaptic glutamate receptors. Table 1 also lists the *Drosophila *gene in which each transposon is inserted, and the mouse homolog of each of those genes (identified by BLASTP against the mouse genome refseq protein database; ).

A definitive test of whether any particular gene is required for glutamate receptor cluster development requires precise excision of each P-element insertion, or transgenic rescue, followed by re-examination of the NMJ at the same developmental stage – a task which is not practical on a genomic scale. However, we performed two broad types of checks to identify potential caveats in our results: expression analysis and complementation tests for lethality.

*Drosophila *glutamate receptors are expressed in neurons and muscles. Thus, any gene required for expression and/or localization of glutamate receptors should be expressed in neurons and/or muscles. The *Drosophila *gene expression database (accessed at: ) describes expression patterns of approximately 3300 different *Drosophila *genes (~24% of the genome), as determined by embryonic in situ cDNA hybridization [22]. Of these 3300 genes, which presumably represent a random 'sampling' of the genome, 2350 (71%) are expressed in the nervous system and/or body wall muscles. The gene expression database contains data for 28 (49%) of the genes identified in our study. 24 (86%) of these genes identified by our screen are expressed in the nervous or muscular systems. 4 (14%) of the genes identified in our screen are annotated as not detectably expressed in the nervous or muscular systems. Thus, most (86%) of the genes identified in our screen have expression patterns consistent with the conclusion that these genes are important for glutamate receptor cluster development, but we did not identify neuronal and muscle genes significantly above the level expected by a random sampling of the genome – possibly because the fraction of genes expressed in neurons and muscles (71%) is quite high to begin with. There may also be cell non autonomous roles for some genes with regard to receptor expression and/or localization.

Although all of the mutants identified in our screens displayed a receptor phenotype, and all of the mutants contain a unique P-element insertion, an important genetic caveat is that the receptor phenotype may not actually be caused by the transposon insertion. For example, the *P*-element mobilizations that generated each of the *P*-element alleles for the gene disruption project may have also created second-site mutations in an unknown locus. Spontaneous mutations can also be easily stabilized and propagated in a stock carrying a balanced lethal mutant chromosome such as those screened here. To estimate the frequency of background mutations in this transposon mutant collection, we performed complementation tests to determine whether the *P*-element insertions failed to complement lethal alleles of the same insertion locus. Of the 57 mutants identified in our screen, 20 were tested for complementation to deficiencies that remove the insertion site. Ten (50%) of the complementation tests failed, suggesting that lethality in half of the mutants may be caused by a mutation other than the *P*-element insertion. These results are consistent with those from a related screen for presynaptic morphology mutants (Liebl FLW, Werner KM, McCabe BD, Featherstone DE: **A Genome-Wide P-element screen for Drosophila synaptogenesis mutants**. M*anuscript in preparation*), which included complementation tests to over 80 genes. Complementation did not appear to be biased for any particular type of gene (in this or the related Liebl et al study). We did not systematically test for complementation of the receptor cluster phenotypes.

## Discussion

In an effort to identify new genes required for glutamate receptor cluster development, we screened lethal transposon insertion mutants for alterations in postsynaptic glutamate receptor clusters. 202 lethal insertion lines, representing insertions in ~1.4% of the genome, were manually dissected and glutamate receptor clusters were examined using immunocytochemistry and confocal microscopy. This screen identified 57 mutations in 56 different loci. Transposon mutageneses are becoming increasingly popular, because a transposon insertion simultaneously mutates and 'tags' a gene. An assumption of all transposon mutagenesis (and subsequent screens) is that the insertion locus is responsible for any observed phenotype. Expression analysis showed that the expression pattern of insertion loci identified in our screen is broadly consistent with that expected from genes involved in glutamate receptor cluster development, but also not compellingly different (86% vs. 71%) from that expected from a random sampling of the genome. Furthermore, complementation tests suggest that approximately one-half of the lethality observed in our screen was not due to disruption of the insertion locus. Thus, it is impossible to definitively say which particular genes are important without extensive validation of each candidate. And even with extensive validation sufficient to confirm that a particular locus is involved, it is difficult to know whether a gene's role in receptor cluster formation is indirect with regard to glutamate receptor cluster formation (i.e. whether a gene product regulates another protein which in turn clusters receptors). Nevertheless, one cannot discount our results without also casting aside the fact that decades of forward genetic screens have successfully identified many genes critical for many different processes [23]. But how does one decide which screen 'hits' to trust, and is there any way to usefully interpret results of forward genetic screens?

Should we exclude from consideration all mutants in which lethality was complemented (and conversely accept all mutants in which lethality failed to be complemented)? This depends on the degree of coupling between two different phenotypes: lethality and abnormal glutamate receptor cluster development. To enrich for mutants with receptor cluster defects, we examined only homozygous lethal mutants, on the assumption that GluRIIA cluster defects and lethality are strongly linked. Indeed, all of the mutants showing severe loss of GluRIIA were embryonic/first instar lethal. Nevertheless, this assumption probably does not hold for all mutants. At the time this screen was initiated, it was assumed that all *Drosophila *NMJ glutamate receptors contained the subunit GluRIIA, and that the viability of GluRIIA null mutants was due to substitution by the alternate subunit GluRIIB [24]. Thus, the presence and localization of GluRIIA was considered an accurate marker for all NMJ receptors. Subsequently, we and others have determined that the *Drosophila *NMJ contains two independently assembled and localized subtypes of postsynaptic glutamate receptors: A-type receptors, that contain the subunit GluRIIA (plus GluRIIC, GluRIID, and GluRIIE, but not GluRIIB), and B-type receptors, which contain the subunit GluRIIB (plus GluRIIC, GluRIID, and GluRIIE, but not GluRIIA) [8-10]. GluRIIA therefore serves as a tag for only one-half to two-thirds (depending on developmental stage; Featherstone, unpublished) of fly NMJ glutamate receptors. Complete elimination of A-type receptors does not result in lethality [24,25]. Conversely, lethality can obviously be caused by many defects other than loss of NMJ glutamate receptors. Since viability and glutamate receptor cluster development may be only loosely coupled, complementation tests for lethality indicate relatively little about the reliability with which our screen identified genes required for proper glutamate receptor cluster formation (although these complementation results do give important insights into the properties of this mutant collection, and transposon mutageneses in general). Similarly, the large fraction of genes expressed in the neuromusculature makes it difficult to judge, based on expression, whether any particular gene has simply been randomly selected. Thus, we are left with uncertainty regarding any particular gene until that gene's role in synaptogenesis can be extensively validated – a prospect that is not practical on a genomic scale. However, the sequencing of several metazoan genomes, along with the realization that the function of homologous proteins tends to be conserved across genomes, opens the possibility of another approach, as demonstrated in our study: 'functional category analysis.'

Functional category analysis involves screening a non-redundant collection of mutants for the phenotype of interest, assigning putative functions bioinformatically, and categorizing the 'hits' by function. The goal of this approach is not to generate a definitive list of individual proteins involved in a process, but to gain insight into the types and relative numbers of proteins required. All of the categories shown in Figure 2, for example, are represented by several mutants. Therefore, even with a 50% accuracy rate (which would be a worst case estimate; the real accuracy is probably substantially higher – see below), the gene function *categories *are likely to be correctly identified. Functional category analysis recognizes that not all hits will be valid, that not all genes will play roles specific to a particular process, and that many proteins are only indirectly required. Functional category analysis reveals unexplored areas of relevant biology and provides a broad roadmap for further study. For example: instead of studying the individual genes identified in Table 1 (which is arguably a hit-and-miss endeavor unlikely to shed much global insight into the process), we plan to focus research toward understanding how RNA regulation and regulated translation are involved in glutamate receptor cluster formation. Similarly, we are directing efforts toward understanding the interactions of receptors with the actin and microtubule-based cytoskeletons. Preliminary results suggest that this approach is insightful and effective [20]; Liebl, Karr, & Featherstone, unpublished observations).

What percentage of the genome is involved in glutamate receptor cluster development? Our results allow this question to be addressed (at least with regard to the *Drosophila *NMJ). If glutamate receptor cluster development and lethality are completely uncoupled, then our results imply that approximately 28% (57/202) of the entire *Drosophila *genome is required for glutamate receptor cluster development. Depending on the percentage of insertions that were too hypomorphic to show qualitatively detectable phenotypes via immunocytochemistry, that percentage could be higher. Glutamate receptor cluster formation and lethality, however, are not unrelated phenotypes. Therefore, 28% is likely to be a gross overestimate. Complete loss of *Drosophila *NMJ glutamate receptors results in paralysis and embryonic lethality [9], and mutations that reduce (but do not eliminate) NMJ glutamate receptors allow hatching, but typically cause larval or pupal lethality [8-10]. In the present study, all severe loss of function phenotypes were associated with embryonic/early larval lethality. If all GluRIIA cluster mutants are lethal, then mutants with normal glutamate receptor clusters would have been eliminated in the selection for lethality early in the screen (indeed, this was the rationale for this step). In this case, our results suggest that approximately 2.6% (57/2185) of the genome is required for glutamate receptor cluster formation. This estimate, however, is also likely to be flawed; although severe disruptions in glutamate receptor cluster formation cause lethality, not all lethality is likely due to disruptions in glutamate receptor cluster formation. Mutations that trigger loss of GluRIIA are not necessarily lethal [24,25]. Lethality and GluRIIA cluster phenotypes are not absolutely coupled, and therefore 2.6% is likely too low of an estimate.

Given the uncertainties above, we can say only that somewhere between 2.6 and 28% of the fly genome (360–3900 genes) is required for NMJ glutamate receptor cluster formation. This is a wide range, which in any case represents a surprisingly large number of genes. Is this reasonable? Do all of these genes represent specific machinery for glutamate receptor cluster expression and clustering? To answer these questions, it is helpful to consider the types of genes identified in the screen. As noted earlier, the role of each individual gene needs to be validated before placing too much emphasis on any particular protein's role. But a general discussion of the genes implicated is helpful for evaluation of the general results.

In support of the idea that our screen correctly identified genes required for glutamate receptor cluster formation, some of the types of genes identified in our screen have been previously identified as important for postsynaptic development. Polo, for example, was identified in our screen, and mammalian polo-like kinases are receiving increasing attention as important players in synapse development [26]. We also identified several other kinases and phosphatases. Activation of the mitogen activated protein kinase (MAPK) pathway facilitates AMPAR surface expression [27,28], and interaction of AMPAR GluR2 subunits with GRIP and PICK1 is dependent upon the phosphorylation of the GluR2 subunit [29,30]. Our screen also isolated mutants in two different ubiquitin ligases. Consistent with this, ubiquitination is known to regulate glutamate receptor number and synapse development [6,31]. Our screen identified a fly neuroligin family member, and mammalian neuroligins were recently implicated in postsynaptic development [32].

One of the largest groups of genes identified in our screen encodes proteins that comprise or regulate the actin and/or microtubule cytoskeletons. Glutamate receptors, like many other proteins, are thought to be transported along microtubules, and anchored to the synaptic actin cytoskeleton [33-35]. In support of this, we've recently found that GluRIIA-containing receptors are specifically linked to the actin cytoskeleton via the 4.1 homolog coracle, which interacts directly with GluRIIA [20]. We've also found that regulation of synaptic microtubules affects fly NMJ glutamate receptor cluster development (see below). The cytoskeleton might not only be important for receptor protein localization; trafficking and localization of synapse-specific mRNAs (see below) probably also relies on the cytoskeleton. Consistent with this, untranslated regions of GluRIIA appear to be required for proper synaptic receptor localization in the fly NMJ (Karr & Featherstone, unpublished).

Other types of genes identified by the screen are consistent with what one might expect. For example, many of the genes identified in our screen are involved in gene expression or protein translation. Mutation of a transcription factor or component of the translation machinery would be expected to disrupt many downstream things, including production of the cellular machinery required for postsynaptic development. Since our screen specifically excluded mutants which did not develop to the later stages of embryogenesis, and which did not form neuromuscular junctions, our screen may have highlighted components of pathways specific for a subset of cell differentiation steps that includes glutamate receptor cluster formation. Some of this machinery is possibly localized to the synapse. *Drosophila *GluRIIA mRNA is localized to the NMJ and locally translated [36]. Mammalian glutamate receptors may also rely on local translation and editing for surface expression [37-39]. Preliminary results also suggest that *Drosophila *NMJ glutamate receptor cluster formation depends on a burst of receptor subunit transcription that follows contact between pre and postsynaptice cells (Karr & Featherstone, unpublished).

Our screen also revealed some important surprises. For example, we identified insertions in two different putative mucin-encoding genes. Mammalian mucins are secreted glycoproteins that are widely implicated in tumor cell adhesion but have no previously identified role in the nervous system [40]. Are these false positives or important insights into postsynaptic development? Interestingly, the 'mucin' genes identified in our screen are also similar to the *C. elegans *polycystin gene *lov-1*, which is localized to the ciliated sensory endings of dendrites required for male mating behavior, where it may be critical for regulating localization of other transmembrane proteins [41,42]. Our screen also identified the exocyst protein sec-8. Sec-8 has recently been implicated in NMDA receptor trafficking but has not been shown to regulate non-NMDA receptor localization, and the mechanism by which it (and other sec proteins) functions remains unclear [43,44]. We are particularly interested in validating and studying the most surprising candidates from out screen, and have done so for sec 8. This work (Liebl FLW, Chen K, Karr J, Sheng Q, Featherstone DE: **Altered Synapse Development in *Drosophila* Sec 8 Mutants**. M*anuscript in preparation*, and Liebl & Featherstone, unpublished) reveals that *Drosophila *sec 8 regulates the synaptic microtubule cytoskeleton to facilitate transmembrane protein localization (Thus the inclusion of sec 8 as a 'cytoskeletal regulator' in Table 1). Thus, even the surprises identified by our screen are, so far, apparently reasonable. But note that even validated candidates, such as sec 8, may not work directly or specifically on glutamate receptors.

Approximately 12% of the genes identified in our screen encode proteins with unknown or novel function. A better understanding of these genes is important for understanding both those protein families and synaptogenesis – assuming these proteins are really regulators of receptor cluster formation. Even with a 50% success rate in identifying genes involved in receptor cluster formation – and the arguments and data above suggest the success rate was much higher – it is clear that novel genes encode a large fraction of proteins required for glutamate receptor cluster development in the fly NMJ. Therefore, major unexplored areas apparently may exist with regard to postsynaptic development. Interestingly, our screen did not isolate any PDZ-domain or MAGUK proteins, which are widely regarded as essential trafficking and scaffold proteins at mammalian glutamatergic synapses [45]. However, this may not reflect a complete lack of importance for these proteins in fly NMJ glutamate receptor localization. *Drosophila *discs-large (DLG) is the sole fly representative of the mammalian DLG/SAP 97/SAP102/PSD-95 protein family; DLG is important for formation of fly NMJ glutamate receptor clusters that contain the subunit GluRIIB, but not those containing GluRIIA [11]. Because we screened only for alterations in GluRIIA immunoreactivity, we would not have isolated DLG mutants. However, our results do support the idea that PDZ proteins are not predominant components of the glutamate receptor localization/stabilization machinery in *Drosophila *NMJs.

Many of the genes implicated by our screen probably do not work directly or specifically. Kinases only have to regulate something that in turn regulates receptor cluster formation. Transcription and translation factors probably control expression of multiple proteins required for PSD formation. Given the fact that many proteins definitively required for glutamate receptor cluster formation may not work directly, it is reasonable that a large percentage of the genome appears to be required. In other words, it is not difficult to identify proteins required for receptor cluster development, and we feel that the demonstration of such by itself does not give real insight into receptor cluster formation. An alternative approach, which we call 'functional category analysis' and introduce here, is to focus on the *types *of genes identified rather than the identities of individual genes, in an effort to gain larger insights into the entire process. Subsequent work can then be directed at investigating the processes represented by these gene types, rather than validation of individual genes that may or may not work directly or be applicable to other synapses and organisms. For example, many microtubule and actin regulators were identified in our screen. The target for these proteins, obviously, is likely to be cytoskeletal proteins instead of receptors. But their identification tells which types of cytoskeleton might be important for glutamate receptor cluster formation. Using these clues, we subsequently determined that microtubules are important for fly glutamate receptor trafficking (Liebl FLW, Chen K, Karr J, Sheng Q, Featherstone DE: **Altered Synapse Development in *Drosophila *Sec 8 Mutants**. M*anuscript in preparation*, and Liebl & Featherstone, unpublished), and that A-type receptors are anchored via the 4.1 protein coracle to postsynaptic actin [20].

## Conclusion

We identified 57 transposon mutants with qualitative differences in glutamate receptor expression and localization. Mutant gene identities need to be validated despite the fact that mutant genes are tagged. Focus on the types of genes identified ('functional category analysis') may provide more useful insight into the process of glutamate receptor cluster formation, compared to focus on individual genes. Our results suggest that glutamate receptor cluster formation involves cell adhesion and signaling, extensive and relatively specific regulation of gene expression and RNA regulation, the actin and microtubule cytoskeletons, and many novel/unexplored processes such as those involving mucin/polycystin-like proteins and proteins of unknown function.

## Methods

NMJ staining and microscopy was performed as previously described [9,19]. Briefly, animals were dissected and fixed for 30–60 min in Bouin's fixative. Late stage embryos were dechorionated in bleach and then manually devitellinated and dissected. Mouse monoclonal anti-GluRIIA ('8B4D2' Iowa Developmental Studies Hybridoma Bank, Iowa City, IA) was used at 1:100. Fluorescently conjugated anti-HRP (Jackson Immunoresearch Labs, West Grove, PA) was used at 1:100. Goat anti-rabbit or goat anti-mouse fluorescent (FITC or TRITC) secondary antibodies (Jackson Immunoresearch Labs, West Grove, PA) were used at 1:400. Confocal images were obtained using an Olympus FV500 laser-scanning confocal microscope. Image analysis and quantification was performed using ImageJ software.

Complementation analysis was performed by crossing GFP-balanced *P*-element mutant stocks to a balanced stock containing a deficiency that removes the insertion site. The F1 generation of each cross was examined for the presence or absence of adult flies carrying neither balancer chromosome. Thus, *P*-element insertion chromosomes were tested for their capacity to complement the viability of the lethal mutations in the gene carrying the *P*-element insertion.

All *P*-element stocks were obtained from Bloomington Stock Center flystocks.bio.indiana.edu. 'Control' genotypes in all experiments are *w*^*1118*^.

## Authors' contributions

FLWL performed most of the immunocytochemistry and microscopy. Analysis was jointly performed by FLWL and DEF.

## Figures and Tables

**Table 1:** Genes affected in glutamate receptor expression/localization mutants. Complete list of the mutants identified in the screen. Table 1 lists all of the mutants identified in the screen, organized by putative function of the protein encoded by the gene in which the transposon is inserted. The qualitative glutamate receptor phenotype in the *Drosophila *mutants is indicated by an arrow pointing upwards (for an increase in immunocytochemically detectable GluRIIA) or downwards (for a decrease in immunocytochemically detectable GluRIIA). The third column lists the *Drosophila *gene that is mutated by the transposon insertion; superscript letters represent complementation test results: 'f' = transposon insertion failed to complement a deficiency covering the region, and 'c' = insertion complemented a deficiency. Note that the confirmed presence of a background lethal mutation (denoted by 'c') does not indicate whether the NMJ phenotype is also complemented. The fourth column lists the mouse homolog, as determined by best BLAST match to an annotated gene in the *mus musculus *refseq database.

**Allele**	**Phenotype**	**Mutant gene**	**Mouse protein**
**Extracellular matrix**			
*P{SUPor-P}CG18819 [KG01657]*	↓	*CG32814*	mucin
*P{SUPor-P}CG14713 [KG05924]*	↓	*CG14713*	mucin
**Cell adhesion**			
*P{SUPor-P}mys [KG02930]*	↓	*mys*	beta integrin
*P{SUPor-P}KG00212*	↑	*Nrt*	neuroligin
**Cytoskeleton, cytoskeletal regulators, and adaptor proteins**			
*P{SUPor-P}CG10540 [KG02261]*	↓	*CG10540*^*c*^	actin capping protein
*P{SUPor-P}KG01009*	↓	*Tkr*	actinfilin
*P{SUPor-P}pnut [KG00478]*	↑	*pnut*^*f*^	septin
*P{SUPor-P}didum [KG04384]*	↓	*didum*^*c*^	myosin V
*P{SUPor-P}neb [KG05913]*	↓	*neb*^*f*^	kinesin
*P{SUPor-P}KG05547*	↓	*Cdic*	dynein intermediate chain
*P{SUPor-P}CG2095 [KG02723]*	↓	*CG2095*^*f*^	sec 8
*P{SUPor-P}lap [KG06751]*	↓	*lap*	synaptosomal protein 91
*P{SUPor-P}noodle [KG03815]*	↓	*CG3210*	dynamin
**Kinases & Phosphatases**			
*P{SUPor-P}KG06341*	↓	*KP78a*^*f*^	MAP/MT affin.-reg. kinase
*P{SUPor-P}SNF4Aγ [KG00325]*	↓	*SNF4γ*	AMP-activated kinase
*P{SUPor-P}trbl [KG02308]*	↓	*trbl*	AMP-activated kinase
*P{SUPor-P}KG04591*	↑	*CG15072*^*c*^	SNF-like kinase
*P{SUPor-P}KG02006*	↓	*Rheb*	RAS-homolog
*P{SUPor-P}polo *[16-1]	↓	*polo*	polo-like kinase
*P{SUPor-P}KG00853*	↓	*CG10082*^*c*^	inositol hexaphosphate kin.
*P{SUPor-P}CG32666 [KG03058]*	↓	*CG32666*	serine-threonine kinase
*P{SUPor-P}KG00564*	↓	*ia2*	rec. tyrosine phosphatase
*P{SUPor-P}l(1)G0003 [KG02485]*	↓	*l(1)G003*^*f*^	Rab coupling protein
**Ubiquitination**			
*P{SUPor-P}Cbl [KG03080]*	↓	*Cbl*	E3 ubiquitin ligase
*P{GT1}sina [BG02648]*	↓	*sina*	sina (ubiquitin ligase)
**Transporters**			
*P{SUPor-P}CG5802 [KG01634]*	↑	*CG5802*^*f*^	UDP-galactose translocator
*P{SUPor-P}KG02272*	↓	*CG8029*^*f*^	lysosomal H+ ATPase
*P{SUPor-P}Vha44 [KG00915]*	↓	*Vha44*	vacuolar H+ ATPase
**Unknown**			
*P{SUPor-P}oaf [KG03408]*	↑	*oaf*^*c*^	D130038B21 RIKEN cDNA
*P{SUPor-P}KG03591*	↓	*CG32345*	hyp. Protein XP_355833
*P{SUPor-P}cmp44E [KG03925]*	↓	*cmp44E*^*c*^	*'*similar to KIAA0953'
*P{SUPor-P}CG2185 [KG02712]*	↑	*CG2185*	calcium binding P22
*P{SUPor-P}KG00333*	↓	*CG15358*	cd209e antigen
*P{SUPor-P}CG31694 [KG04350]*	↑	*CG31694*^*f*^	interferon development reg.
*P{GT1}l(2)35Di [BG02008]*	↓	*l(2)35Di*^*c*^	[none]
*P{SUPor-P}KG06339*	↓	*[none]*	
**Enzymes**			
*P{SUPor-P}CG4825 [KG06018]*	↓	*CG4825*	phosphatidylserine synth.
*P{SUPor-P}TppII [KG03294]*	↓	*TppII*^*c*^	tripeptidyl peptidase II
**Gene expression & Translation**			
*P{SUPor-P}KG02920*	↓	*hsrω *^*f*^	ladybird homeobox-1-like
*P{SUPor-P}KG02514*	↓	*hbn*	aristaless-rel. homeobox
*P{SUPor-P}KG03852*	↓	*Ntf-2*	nuclear transport factor-2
*P{SUPor-P}Trn-SR [KG04870]*	↓	*CG2848*	transportin 3
*P{GT1}CG10689 [BG01776]*	↓	*CG10689*	DEAH box polypeptide
*P{SUPor-P}CG11107 [KG02727]*	↓	*CG11107*	DEAH box polypeptide
*P{SUPor-P}pnt [KG04968]*	↓	*pnt*	avian leukemia oncogene
*P{SUPor-P}KG00625*	↓	*CG18591*	snrpE
*P{SUPor-P}RpL3 [KG05440]*	↓	*RpL3*^*f*^	ribosomal protein L3
*P{SUPor-P}KG03101*	↓	*Ef2b*	translation elong. factor 2
*P{SUPor-P}crp [KG00953]*	↓	*crp*	transcription factor AP-4
*P{SUPor-P}E2f [KG03332]*	↑	*E2f*	E2f transcription factor
*P{SUPor-P}armi [KG04664]*	↓	*armi*^*c*^	Moloney leukemia virus 10
*P{SUPor-P}vri [KG01220]*	↑	*vri*	interleukin3 reg. nuc. factor
*P{SUPor-P}Kr-h1 [KG00354]*	↓	*Kr-h*	zinc finger protein
*P{SUPor-P}Bgb [KG03779]*	↓	*Bgb*	core binding factor beta
*P{SUPor-P}KG06256*	↓	*Chro*^*c*^	Domino (histone deactyl.)
*P{SUPor-P}Chro [KG03258]*	↓	*Chro*	Domino (histone deactyl.)
*P{SUPor-P}EcR [KG04522]*	↓	*EcR*	nuclear receptor 1, group H
